# Effect of pyrolytic carbon addition on the structural and optical properties of TiO_2_ composite thin films

**DOI:** 10.1038/s41598-025-92543-2

**Published:** 2025-03-08

**Authors:** Katarzyna Wojtasik, Michał Wojtasik, Katarzyna Suchanek, Magdalena Zięba, Paweł Karasiński, Wojciech Pakieła, Grażyna Żak, Wojciech Krasodomski

**Affiliations:** 1 Department of Physics, Faculty of Materials Engineering and Physics, Cracow University of Technology, Podchorążych 1, Kraków, 30-084 Poland; 2 Oil and Gas Institute National Research Institute, Kraków, Poland; 3 Department of Optoelectronics, Faculty of Electrical Engineering, Silesian University of Technology, Krzywoustego 2, Gliwice, 44-100 Poland; 4 Department of Engineering Materials and Biomaterials, Faculty of Mechanical Engineering, Silesian University of Technology, Konarskiego 18a, Gliwice, 44-100 Poland

**Keywords:** TiO_2_ doped C, Thin film, Sol-gel, Dip-coating, Raman spectra, Turquoise hydrogen, Two-dimensional materials, Synthesis and processing

## Abstract

The article deals with the preparation and characterization of titanium dioxide thin films containing pyrolytic carbon as potential UV protection films for photovoltaic devices. The carbon used as an additive was obtained by pyrolysis of methane, the main product of which is turquoise hydrogen, and the carbon is a by-product of the process. The resulting carbon material was characterized by Raman spectroscopy, scanning electron microscopy and energy dispersive spectroscopy. Titanium dioxide/pyrolytic carbon composite thin films were prepared by sol-gel method, followed by dip-coating technique. The sols were examined using the dynamic light scattering method. The optical properties of the composite films, including transmittance, reflectance, energy band gap, Urbach energy, porosity, along with their surface morphology and resistance to UV degradation, were evaluated. The results indicate that incorporation of pyrolytic carbon improves the optical properties of composite thin films compared to the samples without carbon, leading to an increase of about 5% in transmittance in the visible range of spectrum. Microscopic observations confirm the presence of pyrolytic carbon in the films, and surface smoothing is noticeable at higher carbon concentrations. These findings suggest the potential use of composite films as UV-blocking films.

## Introduction

Due to the great public interest in photovoltaic devices, which are currently the most common way of generating electricity from available renewable energy sources, there is a constant need to improve the operation, durability and efficiency of these devices^1,2^. The deleterious effects of UV radiation, which accounts for a small and certain portion of the solar radiation reaching the Earth’s surface, have also been reported. UV radiation causes defects in the surface films of photovoltaic devices and thus contributes to a significant loss of their efficiency^3^. The most popular polymer coating used on photovoltaic panels, consisting of ethylene-vinyl acetate copolymer (EVA), loses its strength, impact resistance and changes colour when exposed to ultraviolet (UV) radiation^4^. For this reason, it is extremely important to develop a multifunctional protective coating for photovoltaic panels, with selective absorption of harmful UV radiation and, at the same time, high transmittance of visible (Vis) radiation.

Available UV absorbers include inorganic materials, such zinc oxide (ZnO)^5^ and organic materials, such 2,2-dihydroxy-4-methoxybenzophenone^6^. One of the most popular UV-absorbing materials is titanium dioxide (TiO_2_). It is characterized by a wide energy gap *E*_*g*_ (*E*_*g*_ = 3.2 eV for bulk structures), chemical stability and non-toxicity^7^. In the form of thin films, it is characterized by high transmittance of electromagnetic radiation in the visible range. The material has been widely studied for its application in photocatalysis^8,9^, as UV photodetectors^10^, passive electrodes in electrochromic windows^11^, gas sensors^12^ and others. Zinai et al. presented structural, optical and surface studies of TiO_2_ coatings on glass substrates, which were characterized by low roughness (2.6–3.8 nm), high refractive index and high *E*_*g*_ values^8^. In turn, Sengwa et al. obtained TiO_2_ nanofluid by dispersing TiO_2_ nanoparticles in glycerine^13^. It was shown that these materials can successfully act as UV-blocking materials. By enriching TiO_2_ with a metallic or non-metallic dopant^9^, its optical properties can be improved. Zhumanova et al. showed that a thin film of europium doped TiO_2_ on a glass substrate effectively blocks UV radiation and additionally causes emission of the visible part of electromagnetic radiation in the red range due to the down conversion effect^14^. Recent literature also reports on the beneficial effect of carbon on the properties of TiO_2_ thin films^9^. TiO_2_ films can be fabricated by physical methods, including: electron beam evaporation^15^, pulsed laser deposition (PLD)^16^, magnetron sputtering^8^ and chemical methods, including: chemical vapor deposition (CVD)^17^, atomic layer deposition (ALD)^18^, chemical pyrolysis spray^19^ and the sol-gel method^9,14,20,21^.

Carbon materials are a popular topic of current research around the world. They are products of many chemical processes. The methane pyrolysis method deserves special attention because it is mainly used to produce turquoise hydrogen^22,23^, while the by-product of this process is carbon. Since hydrogen is expected to become a key low-emission fuel for applications such as transportation in the coming years, the methane pyrolysis method is attracting increasing interest. The biggest advantage of obtaining hydrogen in this process is the lack of the need to sequester CO_2_, which simplifies the process and makes this method economically and ecologically competi-tive compared to other methods of obtaining hydrogen^24^. Moreover, methane pyrolysis is a method that not only reduces CO_2_ emissions, but also produces high-purity hydrogen, which is essential for fuel cell applications. If hydrogen is produced by this method on an industrial scale in the future, large amounts of carbon will be created that can be used. Therefore, the search for new applications of pyrolytic carbon (PyC), in line with the circular economy model, will be a key factor in the success of the commercialization of this technology as a cost-effective method of low-emission hydrogen production. Possible applications of PyC are closely related to the nature, morphology and properties of the product obtained in the process^25–27^. However, the structure of the resulting carbon is influenced by process parameters such as temperature and the type of catalyst used^28^. For example, nickel-based catalysts are methane pyrolysis catalysts with a high ability to control the process towards the production of carbon fibres (CF) or carbon nanotubes (CNT) at moderate temperatures (500–700 °C)^27^. Iron-based catalysts are effective in a slightly higher temperature range, but their use also leads to the formation of CNTs^29^. In general, the use of catalysts promotes the formation of amorphous forms of carbon^30^, although carbon with graphitic morphology is commonly found^31^. The use of PyC with mixed mor-phology is not a problem, carbon with this morphology easily becomes a product in the rubber industry^31^. However, amorphous carbons are used, for example, in catalysis^32^. Research on the use of graphitic forms is conducted primarily in electronics^33^.

The main objective of this work is to describe the optical properties, structure and surface morphology of new TiO_2_ thin films with added pyrolytic carbon (TiO2-1, TiO2-2.5, TiO2-5) prepared by sol-gel and dip-coating techniques. The composite films were compared with the reference film of TiO_2_ alone (TiO2-0). The TiO_2_/PyC composite films showed higher transmittance in the Vis range compared to the reference film. We determined the parameters and verified their potential application as UV protective films for photovoltaic devices.

The organization of the work is as follows: "Materials and methods"Sect. presents the materials and characterization methods, and "Results and discussion" Sect. contains the results of the obtained tests along with a discussion. In "Conclusion"Sect. provides a short summary.

## Materials and methods

### Materials

PyC was obtained as a by-product in the methane pyrolysis process, described in Chap. 2.2.1. The remaining reagents and solvents used for the synthesis of the sol were commercial products and were used without prior purification. Tetrabutyl titaniate (TBT, purum) used as a TiO_2_ precursor and diethanolamine (DEA, reagent grade) were purchased from Sigma-Aldrich (Steinheim, Germany). Ethanol (EtOH, 99.8%) was purchased from Avantor Performance Materials (Gliwice, Poland). Tetrapropenyl succinic anhydride (TPSA, pure) was purchased from BASF Polska (Warszawa, Poland). The films were applied onto soda-lime glass microscope slides (Menzel Gläser, Thermo Scientific, Waltham, MA, USA).

In the methane pyrolysis process, our home-made catalyst was used, obtained as described below. 100 g of an aqueous solution containing 49.4 g of cobalt nitrate (purum, Chempur Poland) and 20 g of an aqueous suspension containing 10 g of a silica carrier (SiO_2_, purum, Chempur Poland) were mixed. The resulting suspension was stirred (rotation speed 500 rpm) in a closed vessel at 60˚C for 60 min. The vessel was then opened and the solvent was gently evaporated until a thick paste suspension was obtained. The obtained samples were dried at 120˚C for 12 h, then the samples were ground and dried again for 12 h at 120˚C. The obtained samples were ground again and calcined for 5 h at 700˚C. Table 1 shows the characteristics of the obtained catalyst.

**Table 1:** The characteristics of the obtained catalyst (XRF - X-Ray fluorescence, BET method - Brunauer-Emmett-Teller method).

Parameter	Value
Cobalt content (XRF method), % mas.	24.1
Specific surface area (BET method), m^2^/g	125.3
Pore size (BET method), nm	10.5
Mesoporosity (BET method), %	99.1

### Methods

#### Production of PyC in the process of methane pyrolysis

**Fig. 1:**
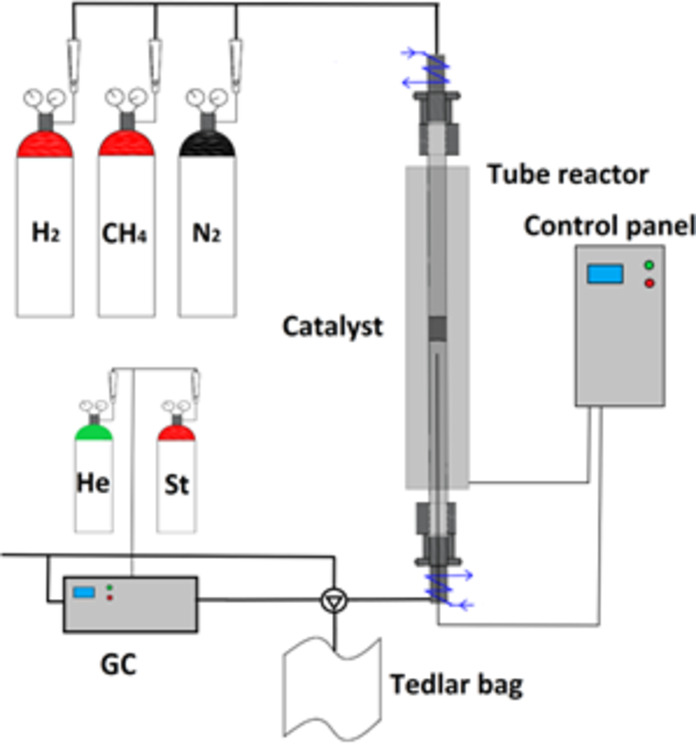
Methane pyrolysis installation (H_2_ – hydrogen, CH_4_ – methane, N_2_ – nitrogen, He – helium, carrier gas, St – reference gas mixtures, GC – gas chromatograph, Tedlar bag – bag to collect gas to analysis.

Methane pyrolysis (Air Product, methane purity: 99.99%) was performed in a quartz reactor with a cylindrical shape, 1200 mm long, with an internal diameter of 85 mm and a wall thickness of 4 mm. The pipe was placed in a glass furnace model PRW 120 × 600/110 M with a power of 3.6 kW (Czylok, Poland) (Fig. 1).

The reactor was closed on both sides with heads with sealing and cooling. The reaction and process gas flow was set at 200 Nml/min for nitrogen and 200 Nml/min for methane. The heating rate was set at 15˚C/min. The catalyst (Chap. 2.1) was placed in a layer of quartz wool and activated before the process at a temperature of 1050˚C for 30 min. After activation of the catalyst, the reactor was cooled to 850˚C and the process was carried out at this temperature for 60 min.

#### Sol synthesis and production of TiO_2_/PyC composite thin films

The titania sols were synthetized in similar way as reported in Ref^34^. The sols were synthesized according to the following procedure: 0.035 mol of TBT, i.e. titania precursor, was dissolved in 1 mol of EtOH. Then 0.02 mol of DEA as a stabilizing agent and 0.01 mol of TPSA surfactant were added^35^. Four sols of titania were prepared in this way. One of which sols was left without addition (named as TiO2-0) and the remaining three were enriched with 1 mol% (TiO2-1), 2.5 mol% (TiO2-2.5) and 5 mol% (TiO2-5) of carbon PyC, respectively. The whole mixture was mixed in closed vessels on a magnetic stirrer at a temperature of 50 °C. After synthesis, the sol was left at room temperature for 24 h.

Films from prepared sols were deposited on soda-lime glass substrates using the dip-coating technique. Before applying the sol, the substrates were mechanically cleaned according to procedures described previously^21^. The application was carried out at a constant withdrawal speed of the substrate from the sol of about 5.40 cm/min. Then the films were heated at a temperature of 485 °C for 1 h. In each case, a durable coating was obtained, without any visible damage or cracks, and constituted a transparent film.

#### Product characterization methods

The determination of the content of methane, hydrogen and the sum of C2 + C3 hydrocarbons (alkanes with two and three carbon atoms in molecule) in post-pyrolysis gases was made using a gas chromatograph (SRI model 8610 C) equipped with a valve-loop dispenser and two thermal-conductivity detectors: thermal conductivity detector (TCD) and helium detector (HID). The chromatograph thermostat operated in isothermal conditions at a temperature of 180˚C, with a flow of helium and nitrogen carrier gas of 10 ml/min. The system was calibrated based on reference gas mixtures (Multax s.c) and single gas patterns. The response coefficients of the TCD detector were determined for individual gases. These coefficients were used to calculate the composition of the tested gas samples originating from the methane pyrolysis process. The post-process gas was characterized online by a coupled chromatograph. The system was additionally equipped with the ability to collect a sample into a Tedlar bag for more intensive analysis.

PyC was imaged using the FEI Quanta 200 FEG scanning electron microscope (SEM) with an energy dispersive X-ray spectrometer (EDX). SEM imaging and EDX analyses were performed at two magnifications in the range (100x – 5000x). The structure information of carbon sample and carbon-containing titanium oxide samples was determined with Raman spectrometer (Almega XR of Thermo Electron Corp.). The excitation light wavelength was 532 nm. Data was recorded in the spectral range from approx. 100 cm^− 1^ up to 4000 cm^− 1^ and with the spectral resolution of 2 cm^− 1^. The sampling depth was approximately 0.7 μm for a 532 nm laser beam. Consequently, meas-urements were carried out on the as-prepared PyC specimens and on a bulk TiO2-0, TiO2-1, TiO2-2.5, TiO2-5 samples with a micrometer size. Prior to the actual measurements, the spectrum of the polystyrene plate was collected for Raman wavenumber calibration. All spectra were normalized, and baseline correction was performed.

The sols were characterized by dynamic light scattering (DLS) using a Zetasizer Nano ZS (Malvern Instruments, Worcestershire, UK) in disposable PMMA (poly(methacrylic acid methyl ester) cuvettes. The hydrodynamic diameters were calculated from the correlation functions using the Malvern Nanosizer Software.

The thicknesses and refractive indices of the produced films were determined using monochromatic ellipsometer SENTECH SE400 (model 2003, *λ* = 632.8 nm, Berlin, Germany). Transmittance, reflectance and absorption characteristics were recorded using UV-Vis AvaSpec-ULS2048LTEC Spectrophotometer (Avantes, Apeldoorn, The Netherlands). In the reflectance measurements the Lab-grade Reflection Probes QR400-7-SR (Ocean Optics, Orlando, FL, USA) was used. In the dip-coating technique, it is characteristic that layers are deposited on both sides of a glass substrate. To carry out reflectance measurements, one of these TiO_2_ films was locally removed and the substrate in this place was matted out. All measurements were performed in the wavelength range 200–1100 nm at room temperature, using Ava-Light-DH-S-BAL (Avantes, Apeldoorn, The Netherlands) as a light source.

Surface morphology of thin films were imaged by SEM using SEM Supra 35 (Zeiss, Oberkochen, Germany). The observations were performed in the secondary electron (SE) and backscattered electron (BSE) with InLens mode (objective lens). The films were observed at magnifications of 15 × 10^3^, 100 × 10^3^ and 250 × 10^3^ at an accelerating voltage of 8–10 kV. The chemical composition of the films was investigated using energy dispersive spectroscopy (EDS) UltraDry EDS Detector (Thermo Fisher Scientific EDS, Waltham, MA, USA). Measurements of the chemical composition were carried out with accelerating voltage of 8 kV. The working distance (WD) for BSE imaging was 14 mm according to EDS calibration. Apertures of 20 and 30 μm were used for the analysis. The results of the chemical composition were corrected using the Phi-Rho‐Z method.

## Results and discussion

### Methane pyrolysis

Table 2 contains the results of methane conversion and the efficiency and selectivity of the process calculated according to formulas (1), (2), (3).

1 $$\:Conversion,\:\%=\frac{products\:conc.}{substrate\:conc.\:}\bullet\:100\%$$

2 $$\:Efficiency,\:\%=\frac{hydrogen\:conc.}{substrate\:conc.}\:\bullet\:100\%$$

3 $$\:Selectivity,\:\%=\frac{Efficiency}{Conversion}\:\bullet\:100\%$$

**Table 2:** The obtained level of methane conversion and the efficiency and selectivity of the methane pyrolysis process.

Parameters	Value
Methane conversion, %	55.5
Process efficiency, %	53.0
Process selectivity, %	95.7

The reported methane conversion in pyrolysis catalysed by cobalt compounds is very diverse, ranging from 17–20%^36,37^ similar to values obtained by other authors^37–39^ and reaching up to 80% in some studies^29,40^. The selectivity of the pyrolysis process with the use of cobalt was estimated in^36,41^ and ranged from 69 to 84%, depending on the composition of the catalyst. The results obtained in this studies are consistent with the previous findings, and in terms of the selectivity of hydrogen production, they can be considered very satisfactory.

Carbon with a characteristic flake structure and silver colour was formed on the reactor walls. Small amounts of carbon in the form of black powder were formed in the area where the catalyst was placed and on its surface.

**Fig. 2:**
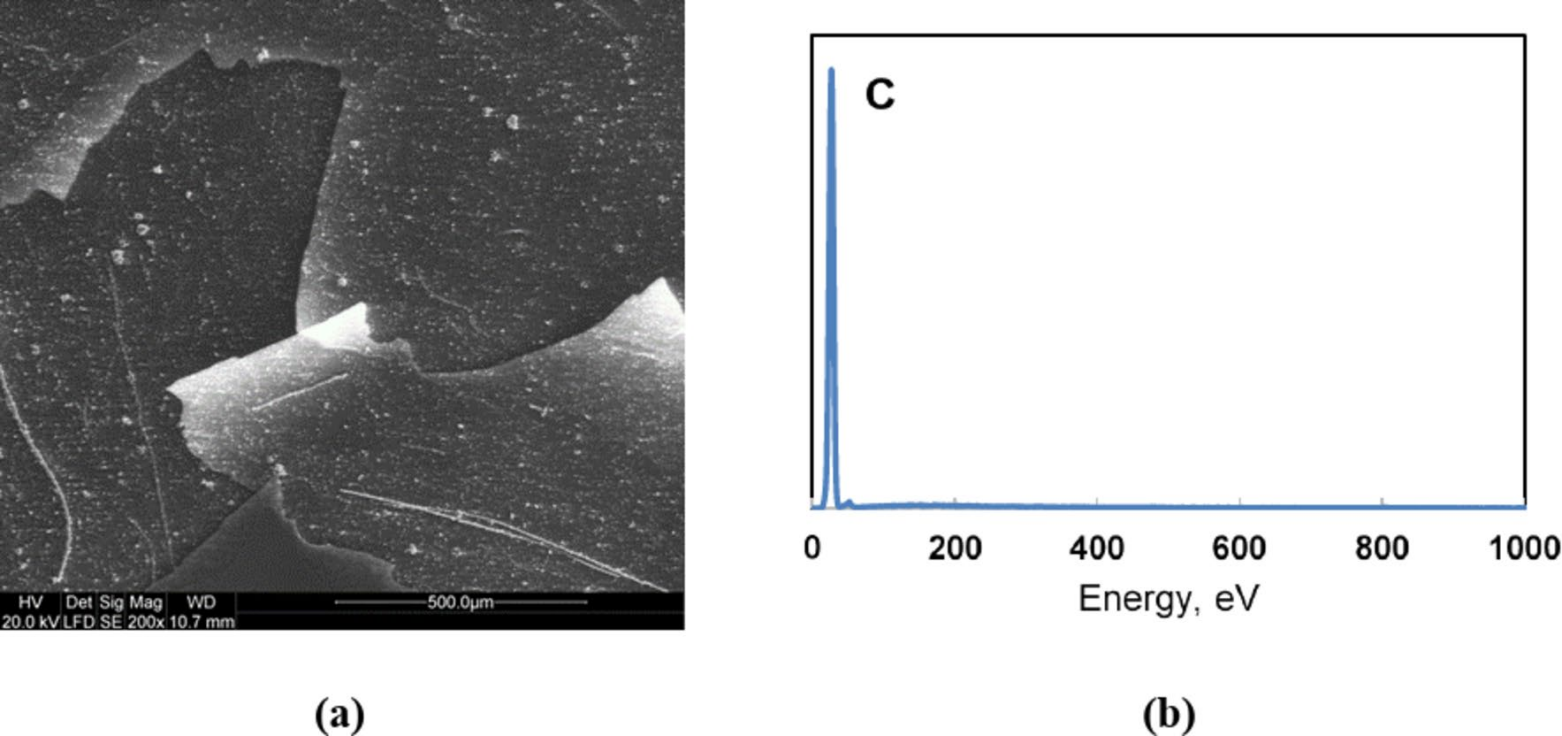
SEM images (**a**) and EDX analysis (**b**) for carbon produced in the methane pyrolysis process

Figure 2 shows images of carbon obtained in the pyrolysis process, recorded in SEM with EDX.

The obtained enlarged images of pyrolytic carbon showed sheet-like carbon structures of small thickness, difficult to estimate in scanning microscopy. On the surfaces of flat carbon structures, small clusters of spherical particles with diameters of approximately 1 μm and numerous fibre-like formations with small diameters could be observed. These structures were probably amorphous forms of carbon (amorphous fibrous forms and “carbon black”) formed in parallel in the process. To support this thesis, an EDX spectrum taken in the areas presented in the SEM photographs was presented, which shows that the composition of the tested material contains only the element carbon.

**Fig. 3:**
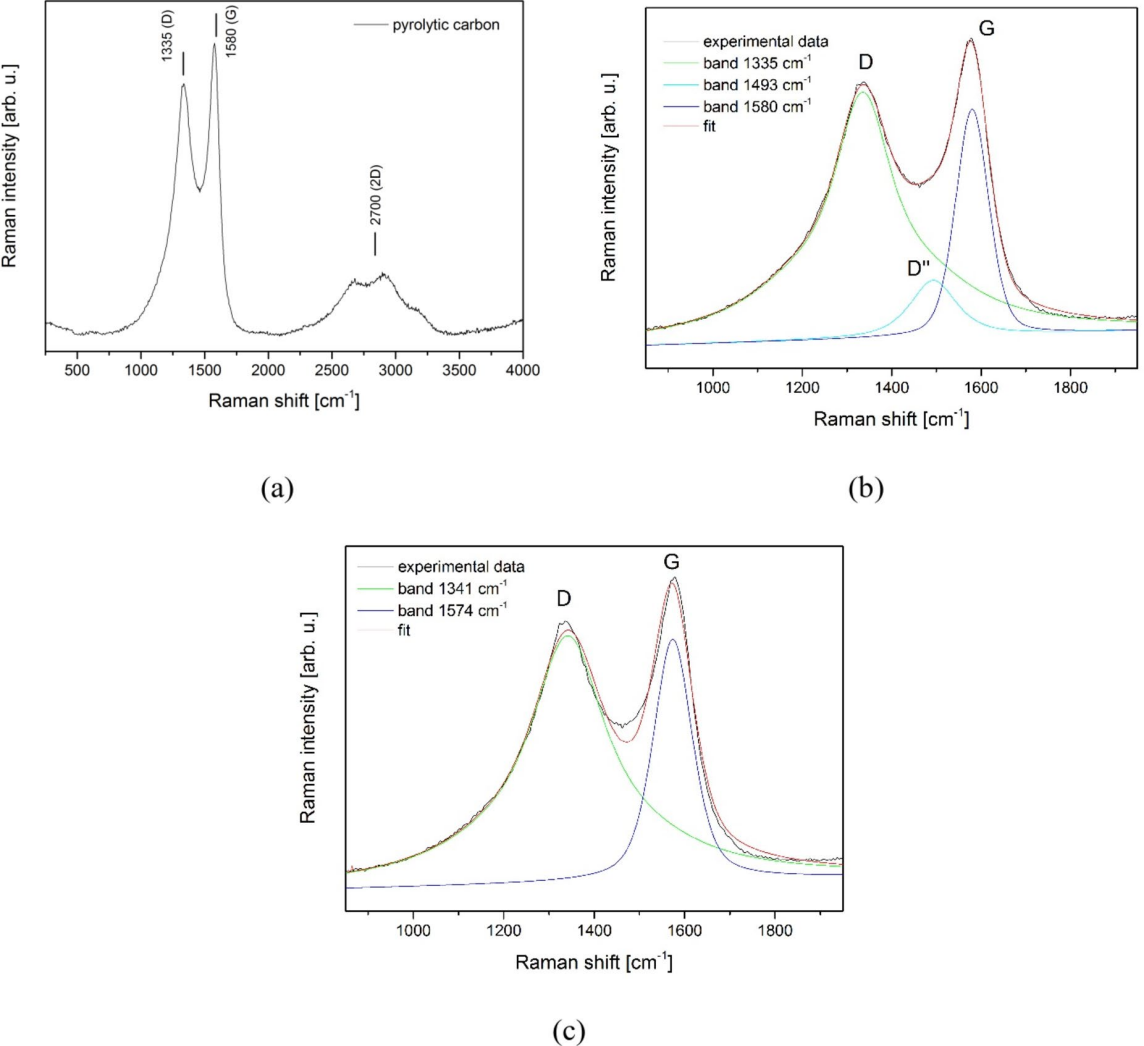
The Raman spectrum of pyrolytic carbon (**a**), three-component deconvolution of the Raman spectrum of PyC (**b**) and two-component deconvolution of the Raman spectrum of PyC (**c**).

Subsequently, we focused on examining an as-prepared carbon specimen through the application of Raman spectroscopy. The representative spectrum obtained is depicted in Fig. 3a.

In general, all forms of carbon exhibit common features in their Raman spectra in the range of 800–2000 cm⁻¹, including the so-called G and D bands, which appear around 1560 and 1360 cm⁻¹ when excited with visible light. The G band has E₂_g_ symmetry and arises from the stretching of all pairs of sp^2^ atoms in both rings and chains. The D band is related to the A₁_g_ breathing mode and results from the breathing modes of sp² atoms in rings^42,43^. At higher wavenumbers, second-order bands also appear, specifically the 2D bands around ∼2700 cm⁻¹^44^.

The spectrum of the PyC sample, depicted in Fig. 3a, reveals a characteristic structure of pyrolyzed carbon, featuring a broad D band (1335 cm^-1^) and a slightly narrower G band (1580 cm^-1^) that partially overlap. The presence of the G band indicates the existence of graphitic carbon, while the D band is associated with disordered (amorphous) carbon in the PyC samples^45^. Additionally, in the range 2250–3250 cm^-1^, D peak overtone (2D) as well as peaks assigned to mode combinations are observed^46^. To analyse this spectrum, we fitted a theoretical curve using a mixed Gaussian and Lorentzian profile. As noted by Ferrari et al., various disordered carbon forms exhibit not only prominent G and D bands but also weaker modulations around 1100–1200 cm⁻¹ and 1400–1500 cm⁻¹^47^. These additional spectral features arise due to structural variations within the carbon matrix, including crystallinity, disorder degree, and oxidation effects^44^. Given this complexity, a multi-component fitting approach is commonly used to analyse the characteristic carbon band in the range of 1000–1800 cm⁻¹^44,47^. In our case, the best fit was achieved using a three-component model, where the intensity of the low-frequency band around 1100–1200 cm⁻¹ was set to zero, as no significant contribution was detected in this region (Fig. 3b). A two-component fit is depicted for comparison in Fig. 3c showing the incomplete overlap between the theoretical curve and the result. The results related to the peak positions are summarized in Table 4. The three-component deconvolution analysis revealed a peak around 1493 cm⁻¹, denoted to as D’’^44^. Its origin has various assignments, with the most recent interpretation linking this band to amorphous lattices. This is based on observations reported by Vollebregt et al., showing a decrease in the intensity of the D″ peak as the crystallinity of the sample increases^48^.

### DLS measurements of sols

**Fig. 4:**
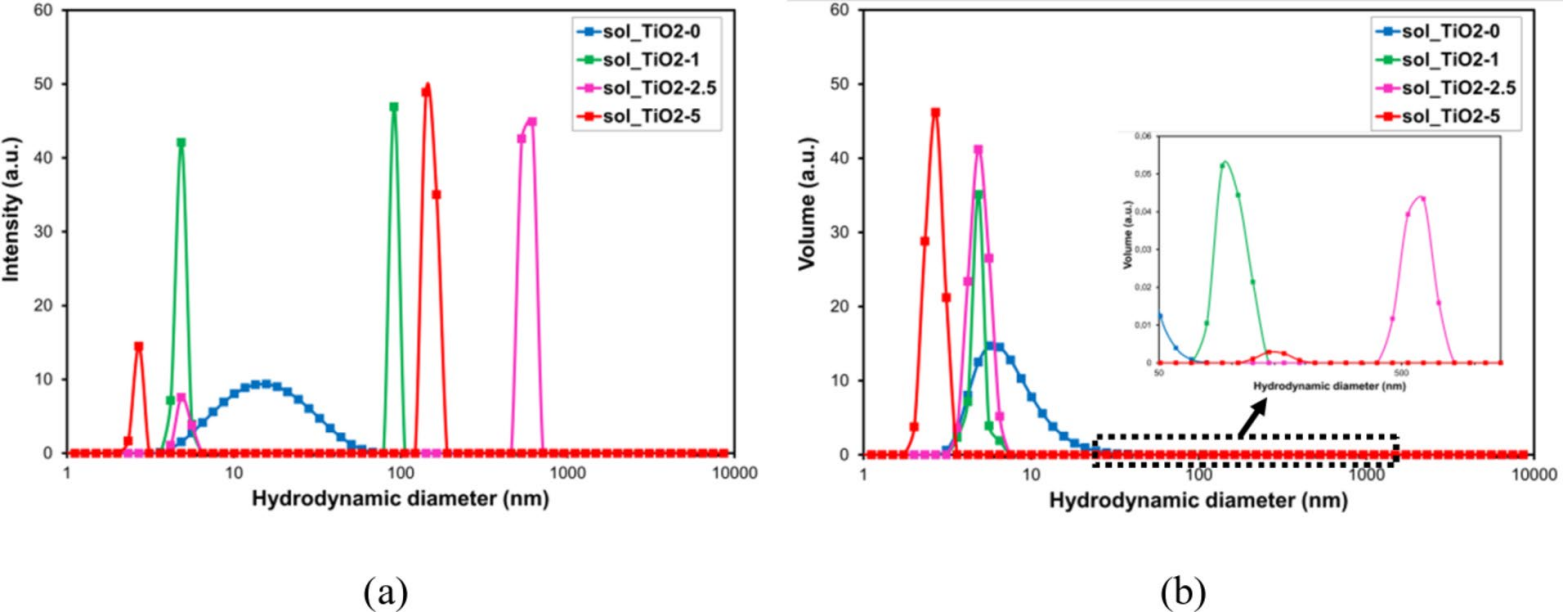
Hydrodynamic diameters of particles in TiO_2_ sols: reference and with PyC addition; size distribution by intensity (**a**) and size distribution by volume (**b**).

Particle size distributions in the sols were determined based on the measurement of the hydrodynamic diameter using the DLS method. Figure 4 shows the results of the obtained measurements.

In the particle size distribution by intensity (Fig. 4a), one broad peak is observed for the TiO2-0 sol with a hydrodynamic diameter of 15 nm, while for sols with PyC addition - two peaks: the first one with a hydrodynamic diameter of 5 nm (TiO2-1, TiO2-2.5) and 3 nm (TiO2-5); the second peak in the range of 91 nm (TiO2-1), 142 nm (TiO2-5) and 615 nm (TiO2-2.5). The obtained results indicate the presence of two types of particles in sols: one with smaller hydrodynamic diameters probably comes from the titania precursor, and the other with larger ones comes from the added carbon. This is also indicated by the particle size distributions in the sols by volume (Fig. 4b), in which we observe the same number of peaks for individual samples. The peak from the titanium precursor for sols without PyC is relatively broad, while the sols with PyC are characterized by a narrow distribution. This is most likely due to the reduction of intermolecular interactions and, as a result, a decrease in the tendency of titania precursors to form agglomerates. The presented thesis is also supported by the effect of shifting the peak maximum towards smaller values (from 15 nm to 3 nm – 5 nm), which is likely caused by the reduction of the thickness of the Debye layer as a result of the decrease of intermolecular forces. Another explanation for the reduction of the hydrodynamic diameters of particles in sols with the addition of PyC may be that this additive affected the rate of hydrolysis and condensation processes occurring in the synthesis of the sol and constitutes a separate solid phase in the system. The differences in the sizes of hydrodynamic diameters for the second fraction (identified as PyC) seem to be random and independent of the PyC concentration and range from about 90 to about 600 nm. This is due to the large variation in the size of the PyC samples used. In order to reflect the actual conditions of the material, the PyC was not specially prepared (ground, homogenized) and the sol was not filtered to eliminate agglomerates and large fractions.

### Films

#### Optical properties

Table 3 presents the basic parameters of the produced TiO_2_ films: reference (TiO2-0) and TiO_2_/PyC composites (TiO2-1, TiO2-2.5, TiO2-5). The films were deposited on soda-lime glass substrate using the dip-coating method at a constant withdrawal speed equal of 5.4 cm/min. The films were annealed at 485 °C for 1 h.

**Table 3:** Basic parameters of the films.

Film	d (nm)	*n* (λ = 632.8 nm)	*P* (%)	E_g_^ind^ (eV)	E_g_^dir^ (eV)	E^U^ (meV)
TiO2-0	51.2	1.970	23.5	3.55	4.09	15.28
TiO2-1	43.6	1.973	23.3	3.56	4.14	14.82
TiO2-2.5	41.0	1.954	20.4	3.53	4.15	15.06
TiO2-5	46.9	1.950	20.5	3.55	4.11	15.07

(*d* – thickness of films, *n* - refractive index of layers, *P* - porosity, *E*_*g*_^*ind*^ – the optical band gap for indirect transitions, *E*_*g*_^*dir*^ - the optical band gap for direct transitions, *E*^*U*^ - Urbach energy).

The obtained results from ellipsometric measurements indicate that the addition of PyC to the TiO_2_ sol affects the thickness and refractive index of the films. All TiO_2_/PyC composite films were characterized by a lower thickness than the reference film (TiO2-0). The lowest thickness was obtained for the TiO_2_ film with 2.5 mol% of PyC (TiO2-2.5). The refractive index values for the TiO_2_/PyC composite films were found to be slightly lower (*n* ~ 1.95) than for the reference film (*n* = 1.970), except for the TiO_2_ film with 1 mol% PyC (TiO2-1), for which the refractive index was *n* = 1.973. It seems that, the addition of PyC in a small amount of 1 mol% does not affect the value of the refractive index.

Using the values of the refractive index *n* of the tested films determined ellipsometrically and the refractive index for anatase - a polymorphic form of TiO_2_ (*n*_*d*_ = 2.52), based on the Lorentz-Lorenz formula^49^, the films surfaces the porosity *P* of the film materials were calculated:

4 $$\:\frac{{n}^{2}-1}{{n}^{2}+2}=\left(1-\frac{P}{100\%}\right)\frac{{n}_{d}^{2}-1}{{n}_{d}^{2}+2}$$.

The obtained results are summarized in Table 3. The highest porosity was determined for the reference film TiO2-0. However, the more PyC added, the lower the porosity of the films. This suggests that PyC is incorporated into the pores of TiO_2_, increasing the compactness of the film material. The incorporation of PyC into TiO_2_ was also observed in Raman spectroscopy measurements (see Chap. 3.3.2.).

**Fig. 5:**
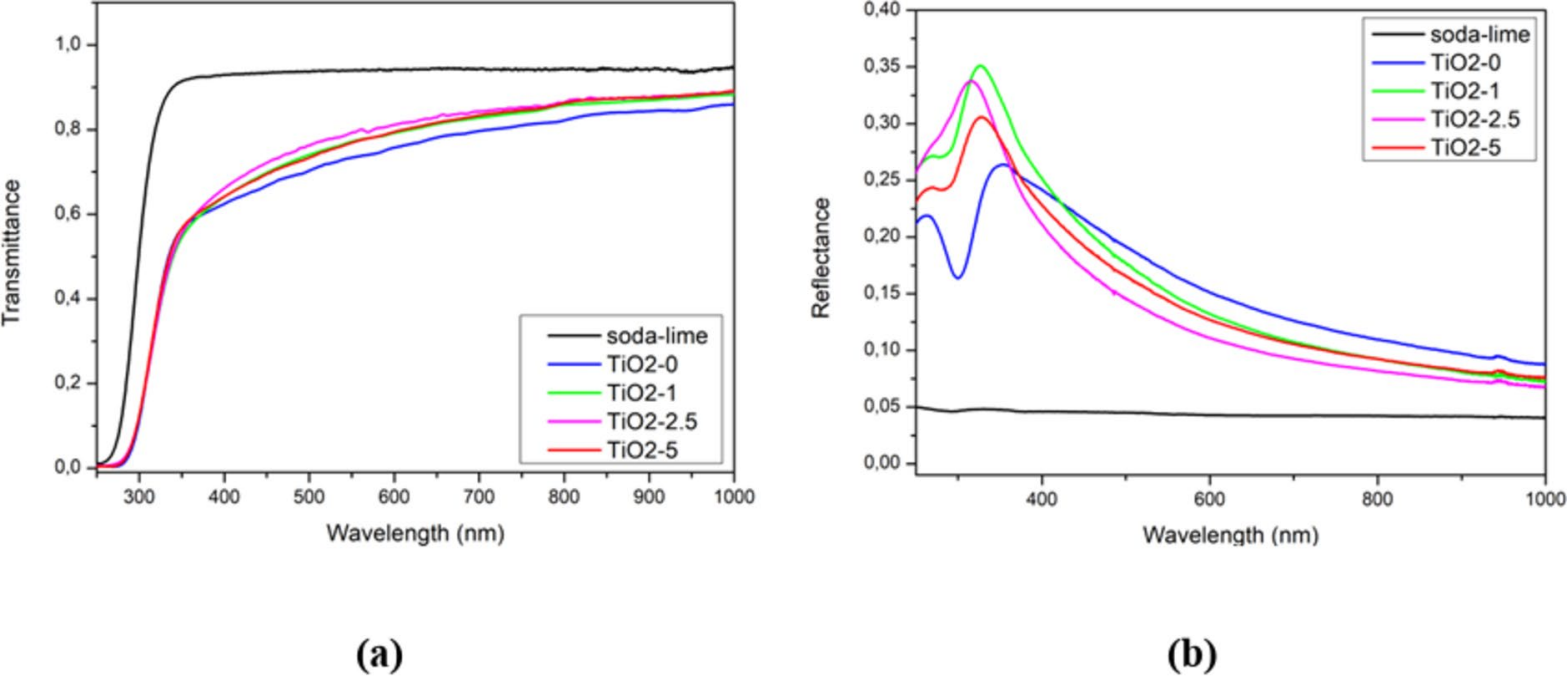
Transmittance (**a**) and reflection (**b**) characteristics of the reference TiO_2_ films and composite films.

Figure 5 shows the transmittance (Fig. 5a) and reflectance characteristics (Fig. 5b) of the produced films: reference (TiO2-0, blue line) and TiO_2_/PyC composites (TiO2-1 - green line, TiO2-2.5 - magenta line, TiO2-5 – red line). The reflectance spectra of all tested TiO_2_ films were recorded at the spot located 15 mm from the top edge of the glass substrate. Both characteristics also include the characteristics for soda-lime glass (black line).

In the transmittance characteristics (Fig. 5a) we do not observe interference maxima and minima, which may be the effect of both small film thicknesses (Table 3) and light decoherence or depolarization processes^50,51^. In the spectral range above 370 nm, all TiO_2_/PyC composite structures showed higher transmittance than the TiO2-0 film. At the same time, in the spectral range above 400 nm, composite films are characterized by lower reflection than the reference film. In the spectral range below 370 nm, the transmission characteristics (Fig. 5a) show decreases in transmission for each structure tested. They are the result of strong absorption of UV light in the TiO_2_ film.

**Fig. 6:**
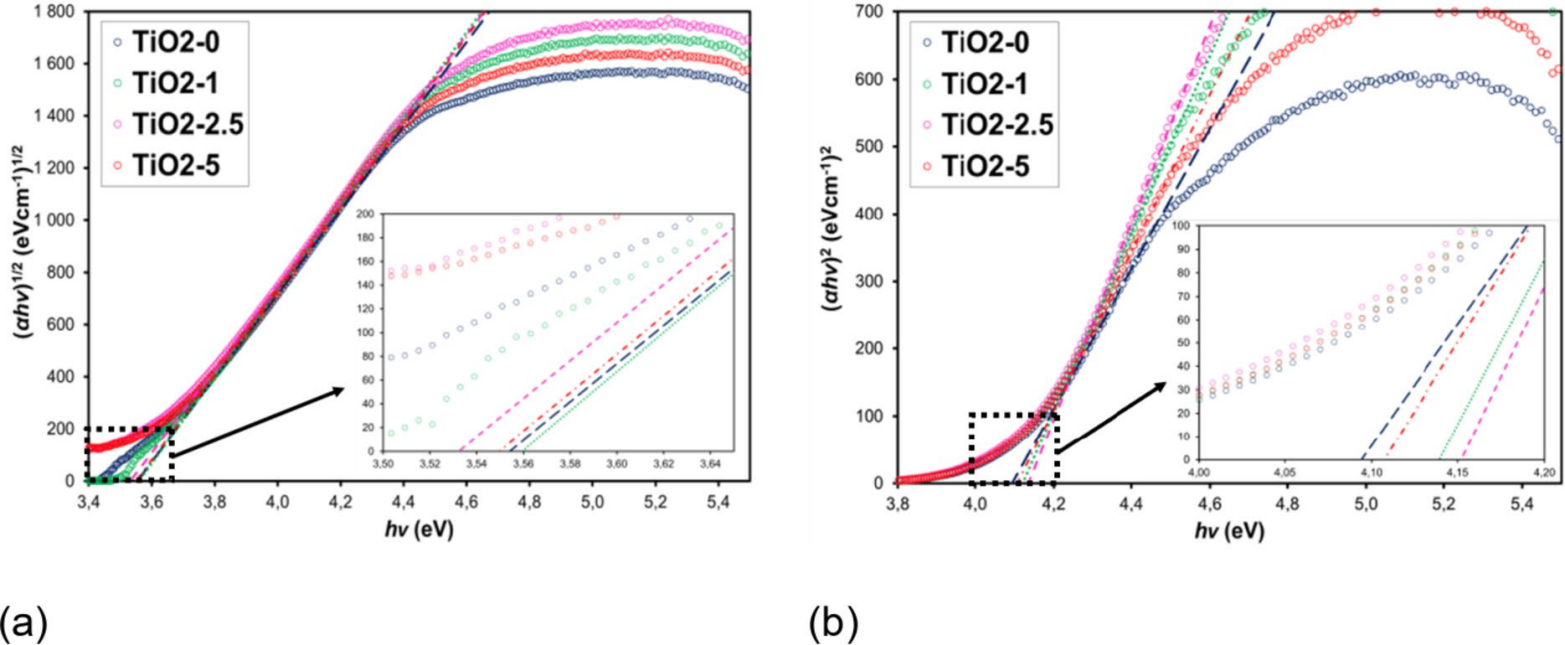
Tauc plots of (*αhν*)^1/2^ as a function of photon energy (*hν*) for indirect optical transitions (**a**) and plots of (*αhν*)^2^ as a function of photon energy (*hν)* for direct optical transitions (**b**).

The optical band gap for indirect and direct transitions of the TiO2-0, TiO2-1, TiO2-2.5 and TiO2-5 films were determined from the Tauc relationship^52^, using their transmittance and reflection characteristics:

5 $$\:\alpha\:h\nu\:=B{\left(h\nu\:-{E}_{g}\right)}^{r}$$

where *α* is the absorption coefficient, *h* is Planck’s constant, *ν* is the frequency of the incident radiation, *B* is the absorption constant, *E*_*g*_ is the width of the material’s energy gap, and *r* determines the nature of the band transition. For TiO_2_, transmittance observations near the absorption edge reveal the occurrence of both indirect and direct interband transitions. For this allowed transitions *r* = ½ for indirect and *r* = 2 for direct optical transitions. The obtained results are summarized in Table 3. The energy gap for indirect transitions (*E*_*g*_^*ind*^) indicates the presence of a crystalline phase in the film material, while the energy gap for direct transitions (*E*_*g*_^*dir*^) is related to the amorphous phase of TiO_2_^50^. Figure 6 shows Tauc plots, i.e. *(αhν)*^*1/2*^ as a function of *(hν)* for indirect (Fig. 6a) and *(αhν)*^*2*^ as a function of *(hν)* for direct optical transitions (Fig. 6b) for TiO_2_/PyC thin films. The optical band gaps were determined by extrapolating the linear part of the experimental curves towards lower energies. The point where the extrapolated line intersects the horizontal axis defines the optical band gap value.

The determined *E*_*g*_^*ind*^ values for TiO_2_ are ​​higher by 0.33–0.36 eV compared to the literature value for bulk TiO_2_ (*E*_*g*_^*bulk*^ = 3.2 eV). This is related to the quantum size effect, which is observed for semiconductor materials^53,54^. Similar *E*_*g*_^*ind*^ values ​​were obtained by Milenov et al.^55^ for TiO_2_:C films of comparable thickness, produced by magnetron sputtering and by Mai et al.^56^ for films produced by sol-gel and spin-coating technique. The obtained results indicate that the addition of PyC does not affect the *E*_*g*_ values ​​for indirect optical transitions, suggesting that PyC has no influence on the crystalline phase in TiO_2_ films. Additionally, the obtained *E*_*g*_ values ​​for direct optical transitions are at least 0.54 eV higher than *E*_*g*_ for indirect optical transitions and show a clear effect of PyC addition on these values ​​(for TiO2-0 *E*_*g*_^*dir*^ = 4.09 eV and for TiO2-2.5 *E*_*g*_^*dir*^ = 4.15 eV). The fact that *E*_*g*_ values ​​for indirect transitions remain unaffected, while the *E*_*g*_ for direct transitions increases with decreasing film thickness (Table 3) suggests that PyC affects the optical properties of the composite. This may indicate a reduced contribution of the amorphous phase in the TiO_2_ structures.

In order to assess the differentiation of the material structure of the films under the influence of added PyC, the Urbach energy was determined^57^. The Urbach energy was determined by plotting in the spectral range close to the first absorption band, the relationship^58^:

6 $$\:\alpha\:\left(h\nu\:\right)={\alpha\:}_{0}\dot\\{exp}^{\left(\frac{h\nu\:}{{E}^{U}}\right)}$$

where *E*^*U*^ is Urbach energy. The obtained values are summarized in Table 3.

The TiO_2_/PyC composite materials exhibit lower *E*^*U*^ value than pure TiO_2_ films. This indicates a reduction in the diversity of the material’s structure. This is supported by earlier findings, where we observed decreased diversity in the composite film structure by analysis of the energy bandgap values. These values showed no change in the crystalline phase of the composite films, while a change of the amorphous phase of the sample was observed with a simultaneous decrease in the film thickness. This effect is additionally reflected in the porosity values ​​(Table 3). Furthermore, the number of defects has probably decreased^59^.

#### Raman spectroscopy

**Fig. 7:**
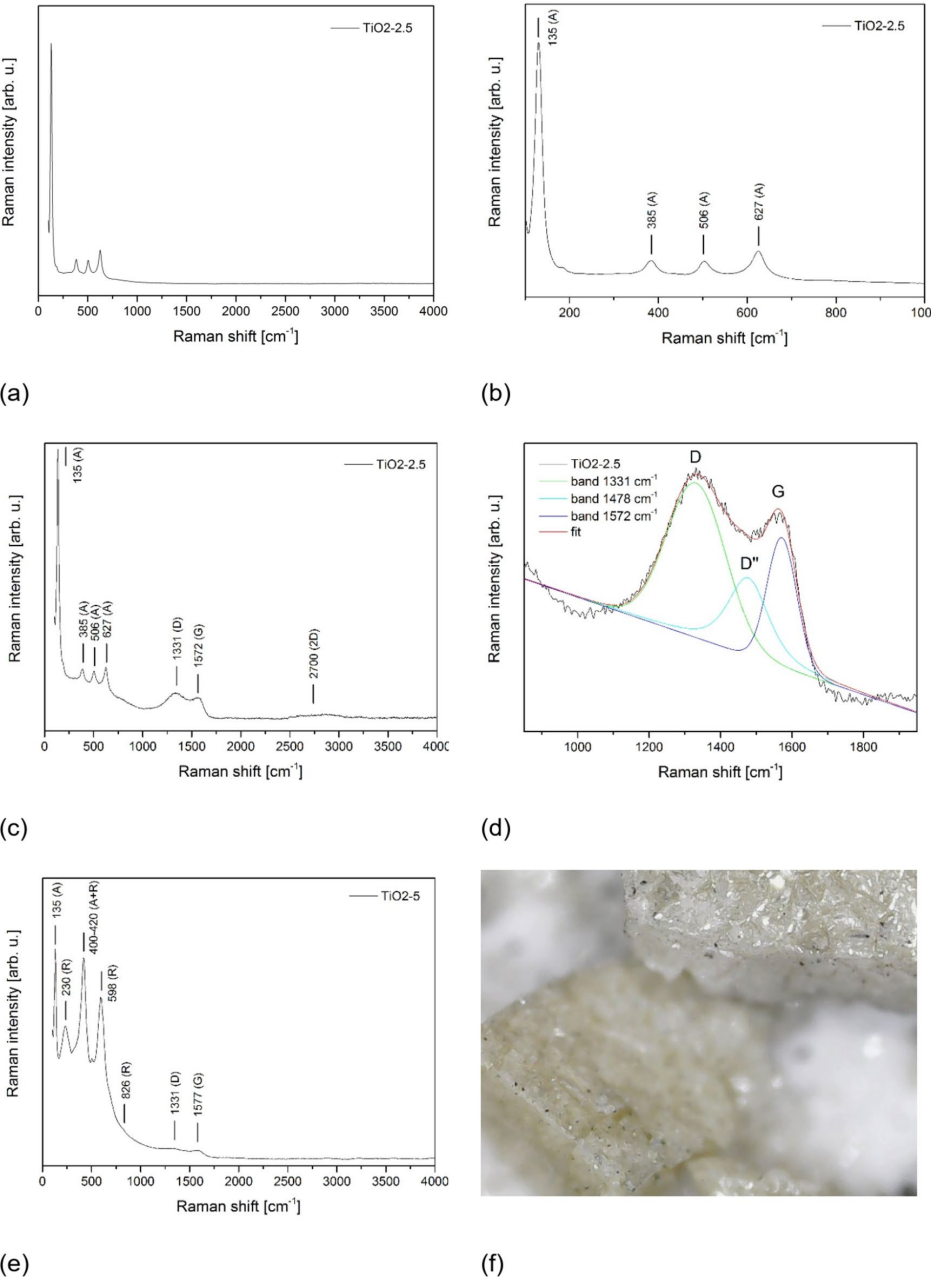
Representative Raman spectrum of TiO2-2.5: a pure titanium oxide site on sample (**a**), a pure titanium oxide site on sample within the spectral range from 100–1000 cm ^–1^(**b**), a carbon-rich site on sample (**c**), three-component deconvolution of the Raman spectrum of bands characteristic for carbon on sample (**d**), and Raman spectrum of a carbon-rich site on sample TiO2-5 (**e**), image from an optical microscope of an example sample examined by Raman spectroscopy (bulk material, prepared from a portion of sol annealed at 485 ˚C) (**f**).

Raman spectra were taken for all carbon-containing titanium oxide samples. In general, the samples were distinguished by regions where the Raman spectrum characteristic for pure crystalline titanium oxide was observed (Fig. 7a, b), as well as places where the Raman spectra contained additional bands characteristic for PyC carbon (Fig. 7c, e). For the sake of clarity, in Fig. 7a-d, we only present the result for the TiO2-2.5 sample, since the same features were observed for the other specimens. Additionally, in the TiO2-5 sample, some extra features were detected (Fig. 7e). Figure 7a, c, e show the spectra in the entire spectral range, while Fig. 7b presents a narrowed range covering only the region of TiO_2_ bands, and Fig. 7d focuses exclusively on the region of carbon bands.

In places indicated the presence of only titanium oxides we see bands characteristic of anatase. Based on the factor group analysis, and assuming site symmetries for Ti and O atoms in the unit cell, there are six Raman active modes for anatase, assigned as follows: A_1g_ (518 cm^− 1^) + B_1g_ (399 cm^− 1^) + B_1g_ (512 cm^− 1^) + E_g_ (144 cm^− 1^) + E_g_ (198 cm^− 1^) + E_g_ (639 cm^− 1^)^60^. The most intense band is centred at 144 cm^− 1^ and it is a particularly sensitive detector of even very small amounts of crystalline TiO_2_ in anatase form. The Raman spectra of TiO2-1, TiO2-2.5 and TiO2-5 show a slight shift of the anatase peaks compared to the literature values, which may indicate the presence of defects in the structure^60^.

In the spectra shown in Fig. 7e, the presence of both titanium oxides and disordered carbon bands was identified, with titanium oxide occurring in anatase and rutile forms. Based on factor group analysis, rutile is expected to exhibit four Raman-active modes at 143 cm⁻¹ (B_1g_), 447 cm⁻¹ (E_g_), 612 cm⁻¹ (A_1g_), and 826 cm⁻¹ (B_2g_)^61^. The weak band observed in spectrum at about 230 cm^− 1^ was assigned to O–O interactions involving three- and four-coordinate oxygen atoms^61^. In the spectral range of 900–1900 cm⁻¹, a three-component deconvolution was performed (Fig. 7d), following the same approach as for pure PyC. The results are summarized in Table 4. The carbon-related bands exhibit features similar to those of a pure PyC specimen (Chap. 3.1), showing the characteristic D and G peaks with a slight modulation corresponding to the D″ band. This suggests that the carbon phase within the sample remains in a state comparable to its form prior to thin films synthesis, indicating existing as a separate phase. Figure 7f shows an optical microscope image of an example sample of TiO_2_/PyC composite obtained by annealing a portion of sol at 485 ˚C subjected to measurements.

**Table 4:** Assignment and position of carbon bands in Raman spectra for different samples.

Band assignment	PyC band position [cm^–1^]	TiO2-1 band position [cm^–1^]	TiO2-2.5 band position [cm^–1^]	TiO2-5 band position [cm^–1^]
D band	1335	1334	1331	1331
Around 1500 cm^–1^	1493	1500	1500	1482
G band	1580	1578	1572	1577

#### Scanning electron microscopy

**Fig. 8:**
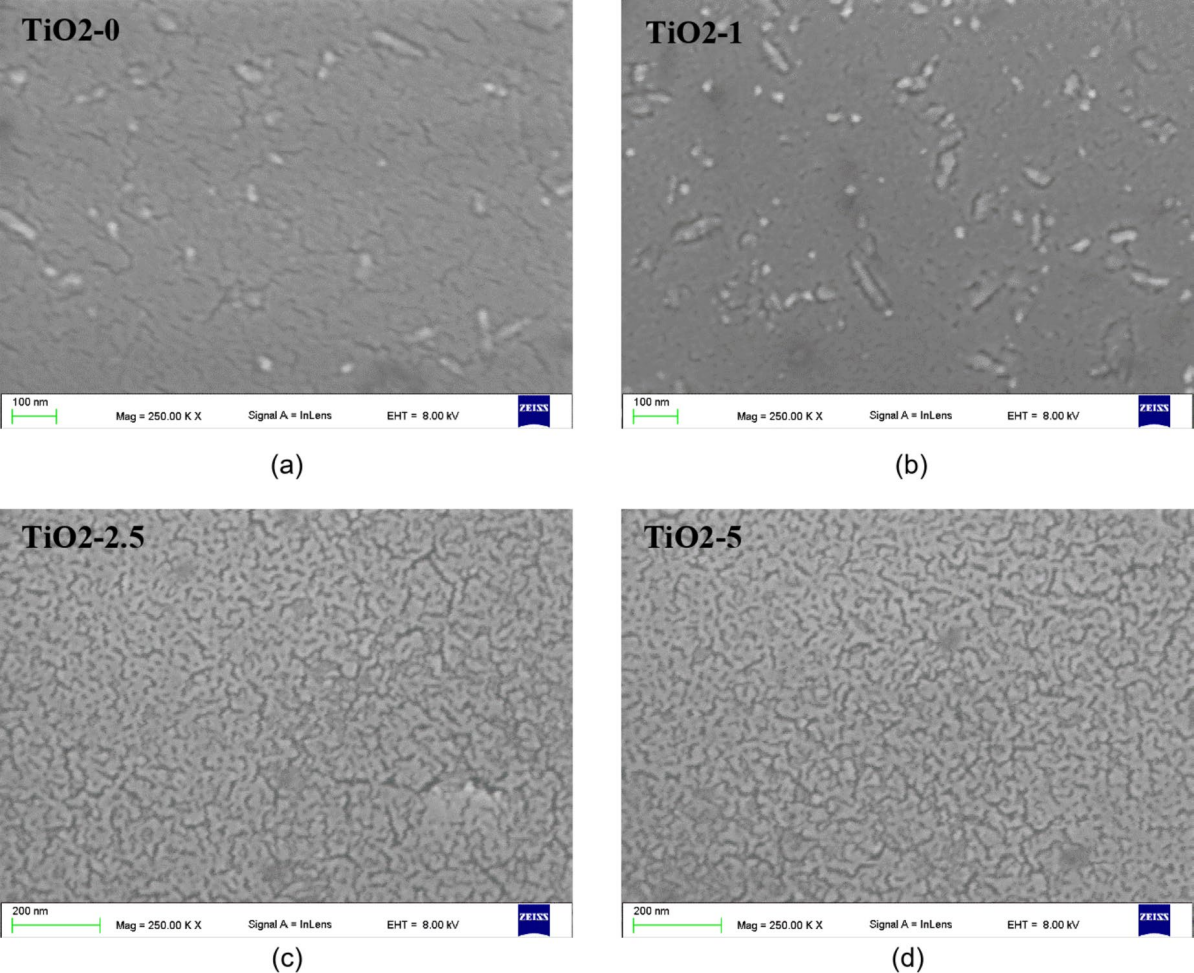
Top view SEM images of the TiO_2_ films (**a**) and TiO_2_/PyC composite films (**b**-**d**).

Figure 8 shows images of the surface of the studied TiO_2_ films obtained at a magnification of 250.00 K× using a scanning electron microscope. Analysis of the obtained images allows us to state that the reference film TiO2-0 (Fig. 8a) is characterized by a uniform surface, without cracks. Single, irregular grains are visible in the film. Similar images for TiO_2_ films annealed at a temperature of about 500 ˚C were observed in our earlier work^21^. The surface of the TiO2-1 film (Fig. 8b) is comparable to that of the reference film (TiO2-0). On the other hand, the images of TiO2-2.5 and TiO2-5 films (Fig. 8c and d), reveal that the TiO_2_ structure become more regular and smaller. The surface of the films is smoother, without cracks and inclusions. For these films, lower porosity values ​​were also obtained than for the reference film (Table 3). Considering that the determined Urbach energy values ​​(Table 3) indicate a higher degree of structural ordering in TiO_2_/PyC composite films than in TiO2-0 it can be concluded that adding an appropriate amount of PyC can improve the surface morphology of the TiO_2_/PyC films. Similar observations regarding the effect of the additive on the surface morphology of TiO_2_ films were observed by Chen et al.^62^.

**Fig. 9:**
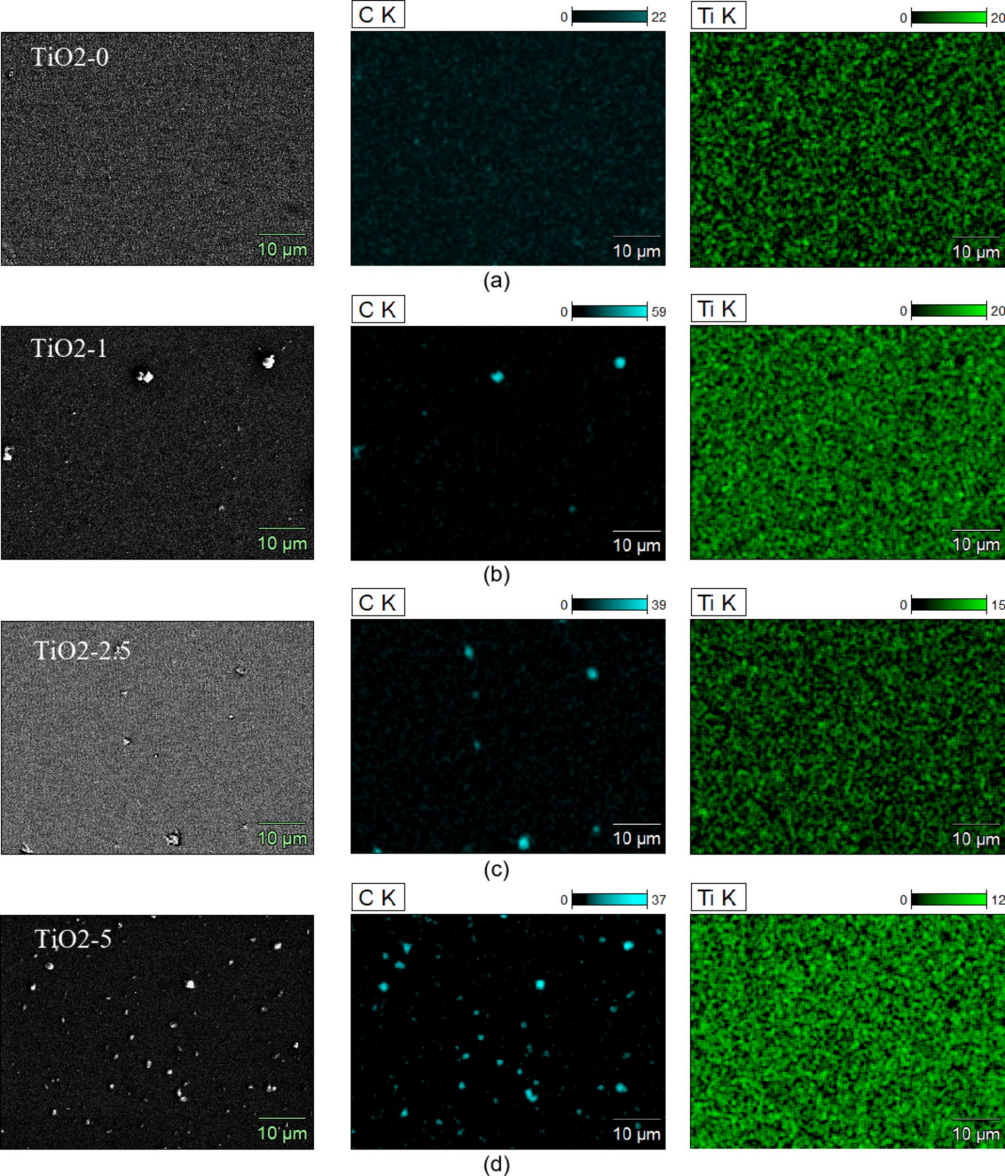
EDS mapping analysis of thin films: TiO2-0 (**a**), TiO2-1 (**b**), TiO2-2.5 (**c**) and TiO2-5 (**d**).

Figure 9 shows SEM-EDS mapping images of the tested films. The first column shows images of the film surfaces at 5000× magnification. The second column shows the distribution of the carbon inclusions on the tested surface (blue colour) and the third column shows the distribution of titanium atoms on the tested surface (green colour). Analysis of the obtained surface images confirms the presence of PyC in all composite films. The images show that PyC in thin films takes the form of random inclusions. An increase in the amount of signal originating from carbon atoms is also observed with the increase in the amount of PyC added.

#### Degradation of films under the UV radiation

**Fig. 10:**
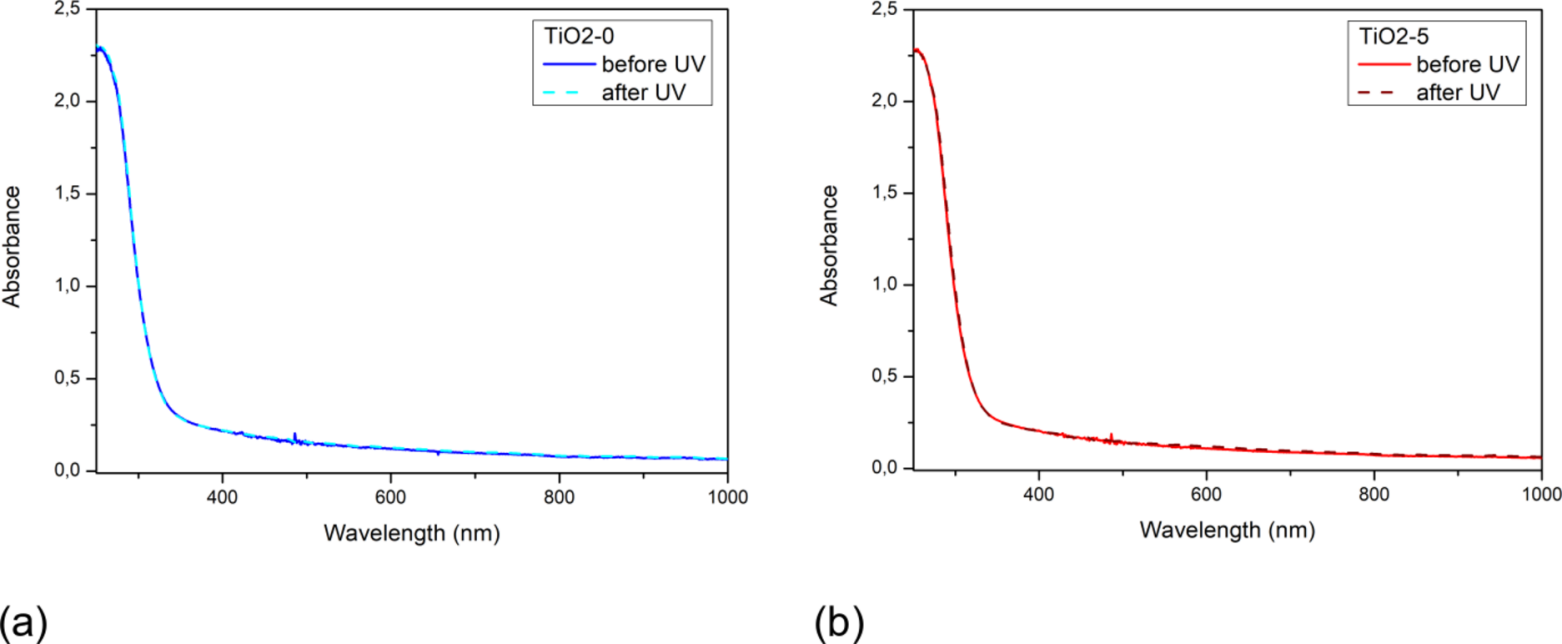
Absorption characteristics of TiO2-0 (**a**) and TiO2-5 (**b**) films before (solid lines) and after irradiation with UV (dash lines).

In order to confirm the durability of the films after exposure to degrading radiation, absorption measurements of the film were performed before and after exposure to UVA radiation (UV diode, *λ* = 365 nm, *Irradiance* 8.6 µW/mm^2^) for 120 min. The reference film (TiO2-0) and TiO_2_/PyC composite films (TiO2-1, TiO2-2.5, TiO2-5) were tested. Figure 10 shows the recorded absorption characteristics for the TiO2-0 film (Fig. 10a) and an example composite film: TiO2-5 (Fig. 10b). For each film, an overlap of spectra taken before and after exposure was observed in the entire measurement range, and below the 370 nm range, a clear absorption peak is observed in each case.

The obtained results clearly show that both the reference and TiO_2_/PyC composite films absorb UV radiation and do not degrade under the influence of this radiation.

## Conclusion

In the present study, we propose a new facile strategy for synthesizing composite TiO_2_ films enriched with pyrolytic carbon (PyC) which exhibit improved optical properties. PyC was obtained using a home-made catalyst (Co/SiO_2_) and through methane pyrolysis, with turquoise hydrogen as the main product and PyC as a by-product. Specimens obtained in the pyrolysis process were characterized by SEM microscopy with EDS and Raman spectroscopy. The results revealed that the PyC structure is predominantly graphitic, with small amounts of amorphous species present as surface inclusions. This was further supported by Raman spectroscopy, where the spectra exhibited features confirming the presence of graphitic carbon and disordered (amorphous) carbon. Pyrolytic carbon was successfully incorporated into titanium dioxide thin films by dip-coating technique. DLS results from sols and SEM images of films confirmed the presence of PyC in both the sols and the TiO_2_/PyC composite films. The composite thin films exhibited 5% higher transmission in the visible range, a smoother surface morphology with more regular grains, reduced porosity and greater structural order (as indicated by lower Urbach energy) compared to reference films. The presence of PyC did not significantly affect the value of the TiO_2_ energy bandgap width for indirect interband transitions. For the reference film TiO2-0 *E*_*g*_^*ind*^ was 3.55 eV and for composite films in the range of 3.53–3.56 eV. The obtained values ​​may indicate that PyC does not alter the crystalline phase of the film. However, a more pronounced effect was observed on the *E*_*g*_ value for direct interband transitions, with an increase from 4.09 eV for TiO2-0 to 4.15 eV for TiO2-2.5, indicating the influence of the amorphous phase in the film. The study of the effect of UV radiation on the degradation of the produced films showed the stability of the film material under such conditions.

We have demonstrated that the methane pyrolysis process, which generates turquoise hydrogen as its primary product, not only serves as a sustainable energy source but also yields valuable by-products such as PyC. This by-product has been shown to enhance the properties of TiO_2_ films, improving their transmission in the visible range, surface smoothness, and structural order.

## Data Availability

The datasets generated during and/or analysed during the current study are available from the corresponding author on reasonable request.
